# HIV testing and associated factors among men (15-64 years) in Eastern Africa: a multilevel analysis using the recent demographic and health survey

**DOI:** 10.1186/s12889-022-14588-6

**Published:** 2022-11-24

**Authors:** Dagnew Getnet Adugna, Misganaw Gebrie Worku

**Affiliations:** Department of Human Anatomy, School of Medicine, College of Medicine and Health Science, University of Gondar, Gondar, Ethiopia

**Keywords:** Multi-level analysis, HIV testing, Men, Associated factors, East Africa

## Abstract

**Background:**

Despite significant efforts made to prevent human immunodeficiency virus (HIV) transmission, its testing coverage among men is still low and remains a major concern in low-income countries, particularly in East Africa. Therefore, this study aimed to determine the prevalence and associated factors of HIV testing among men in Eastern Africa.

**Methods:**

We analyzed secondary data using Demographic and Health Surveys (DHS) drawn from Eastern African countries. Besides, we merged DHS data from eleven Eastern African countries. In this study, we included secondary data from 113, 270 men aged 15-64 years. The outcome variable of this study was “ever been tested for HIV”. Bivariable and multivariable multi-level logistic regression analyses were employed. In the bivariable analysis, variables having a *P*-value of less than 0.2 were selected for multivariable analysis. Lastly, variables with a *P*-value of < 0.05 in the multivariable analysis were declared as a significant factor associated with HIV testing and the adjusted odds ratio (AOR) with the 95% confidence interval (CI) were computed to determine the strength and direction of the association.

**Results:**

The overall prevalence of HIV testing among men in eastern Africa was 60.5% (95% CI: 60.2, 60.7%). In the multivariable multilevel analysis; participant’s older age, being married, increased poverty, HIV knowledge, risky sexual behavior, and being covered by health insurance were positively associated with HIV testing coverage among men. However, men with higher community illiteracy levels, residing in rural settings, age at first sex ≥20 years, and higher stigmatized attitudes towards HIV/AIDS had lower odds of being tested for HIV.

**Conclusion:**

The overall prevalence of HIV testing among men in eastern Africa was relatively higher than the previous studies. The study revealed that age, marital status, residence, age at first sex, community poverty level, community illiteracy level, HIV knowledge, HIV stigma indicator, risky sexual behavior, and health insurance were significantly associated with HIV testing coverage among men. Therefore, all the concerned stakeholders need to develop an integrated strategic plan through providing special attention to the factors that affect the uptake of HIV testing to raise awareness about the importance of HIV testing and to prevent HIV/AIDS transmission.

## Background

Human immunodeficiency virus (HIV) testing and counseling are considered critical steps to the prevention of HIV transmission, treatment, care, and other supportive services [1]. HIV testing is a public health initiative aimed at diagnosing and reducing the spread of HIV [2]. Expanding HIV testing coverage and accessibility to communities with low testing prevalence could help to speed up HIV epidemic control and elimination efforts [3]. Worldwide, remarkable advancements have been made in efforts to fight against HIV and Acquired Immune-Deficiency Syndrome (AIDS). Despite these significant advancements, HIV/AIDS epidemic remains a main global public health problem [4]. In 2018, about 37.9 million people were living with HIV worldwide, with 1.7 million new infections and 770 thousand deaths due to HIV/AIDS [5]. In addition, the highest-burden was observed in Africa, with over 25 million people affected by HIV [4]. In Africa, HIV testing prevalence ranges from 10% in Burkina Faso to 69.9% in Malawi [6, 7]. The prevalence of HIV testing among men in Sub-Saharan Africa is 53.5% [8]. This variation in HIV testing among different regions is due to the difference in lifestyle, cultural beliefs, quality and availability of HIV testing services, and knowledge about HIV/AIDS across different countries [7].

Based on 2015 world health organization (WHO) reports, only 54% of HIV-positive people are aware of their serostatus [9]. Besides, about one-third of adult males get HIV testing services, whereas more than two-thirds of adult females get it, indicating a gender difference in HIV testing rate [9]. Previous studies in Sub-Saharan Africa have also revealed that HIV testing is lower in men than in women [10, 11]. For instance, men (38.3%) have a lower rate of HIV testing than women (47.6%) in Mozambique [11].

According to previous studies carried out in the different regions of the world; marital status, age, level of educational status, region of residence, having multiple sexual partners, wealth index, used condoms, exposure to mass media, and age at first sex were factors significantly associated with HIV testing among men [1, 4, 7, 8, 12–15]. Stigma toward HIV patients, comprehensive HIV knowledge, and risky sexual behavior have also positive associations with HIV testing and counseling [1, 8, 12, 14, 16–18]. Furthermore, family planning discussions with the health workers were significantly associated with the uptake of HIV testing and counseling.

Despite significant efforts made to prevent HIV/AIDS transmission, its testing coverage among men is still low and becomes a major concern in low-income countries, particularly in Eastern Africa [19]. Besides, studies on the prevalence and associated factors of HIV testing among men are limited. Moreover, identifying the associated factors of HIV testing is a very important step to develop and design effective programs, policies, and strategies that target men in Eastern Africa. Therefore, this study aimed to determine the prevalence and associated factors of HIV testing among males in Eastern Africa using recent national HIV Demographic and Health Surveys.

## Methods

### Data sources and study population

This study used secondary data drawn from the most recent Demographic and Health Surveys (DHS) carried out in eleven Eastern Africa Countries (Burundi, Ethiopia, Kenya, Comoros, Madagascar, Malawi, Mozambique, Rwanda, Uganda, Zambia, and Zimbabwe). These datasets were merged to determine the prevalence and associated factors of HIV testing among men in eastern Africa. The DHS is a nationally representative survey that collects data on vital health indicators such as mortality, morbidity, family planning service utilization, fertility, and maternal and child health. Every country’s survey consists of different datasets like men, women, children, birth, and household datasets. For this study, we used the men datasets (individual record files) and we included all men whose age is 15 years and above. Lastly, a total weighted sample of 113, 270 men were included in this study. The survey year and the total weighted sample obtained from every country are shown in Table 1.

**Table 1:** indicates the overall weighted sample of each country

Country	Year of survey	Sample weight	Percent
Burundi	2016/17	7552	6.7
Ethiopia	2016	12,688	11.2
Kenya	2014	12,819	11.3
Comoros	2012	2167	1.9
Madagascar	2008/9	8586	7.6
Malawi	2015/16	7478	6.6
Mozambique	2011	4035	3.6
Rwanda	2014/15	6217	5.5
Uganda	2016	10,672	9.4
Zambia	2018	24,264	21.4
Zimbabwe	2015	16,792	14.8
Total		113,270	100

### Study variables

The outcome variable of this study was “ever been tested for HIV” (a binary outcome variable which was coded as “0” for no and “1” for yes). The independent variables of this study were categorized as individual and community-level variables. The individual-level factors included in this study were: age (categorized as 15-19, 20-29, 30-39, and ≥ 40 years), residence (urban and rural), marital status (never in union, married, living with a partner, and widowed/divorced/separated), educational status (no education, primary education, and secondary or above), age at 1st sex (< 20 and ≥ 20 years), stigmatized attitude towards people with HIV, HIV knowledge, risky sexual behavior, and sex of housed holds. The community-level factors included in this study were: residence, community poverty level, and community illiteracy levels. The community illiteracy level of men was created by aggregating the individual-level variable of men’s educational status by considering the proportion of men in the community that did not have formal education and by grouping this proportion as high and low based on the national median value. Besides, the community poverty level of men was also created by aggregating the individual-level variable men’s wealth index by considering the proportion of men in the community that are poor and by grouping this proportion as high and low based on the national median value.

### Operational definitions

#### HIV knowledge

Participants’ HIV knowledge was assessed based on six questions; three questions related to HIV prevention and three questions related to the modes of HIV transmission. Then, it was categorized into three grades: “low” (if a man respond to at least three questions correctly), “high” (if a man respond 4 to 6 questions correctly), or “comprehensive knowledge” (if a man respond six questions correctly) [20].

#### Risky sexual behavior

This variable was generated based on the 5 questions; had any STI in the last 12 months, genital sore/ulcer in last 12 months, genital discharge in last 12 months, had at least one sexual partner other than the wife in the last 12 months, and multiple lifetime sexual partnerships. These were combined into an index of risky sexual behavior with 3 categories: “no risk” (if the answer is no for five questions), “some risk” (if the answer is yes for only one question), and “high risk” (if the answer is yes for 2-5 questions) [20].

#### HIV stigma index

This variable was created based on 6 questions, which indicate negative attitudes towards people living with HIV / AIDS. Therefore, HIV stigma index was graded as “no stigma” (if we got a score of 6), “low stigma” (if we got a score of four to five), “moderate stigma” (if we got a score of two to three), and “high stigma” (if we got only score one) [20].

### Data management and analysis

STATA version 14 software was used for data recoding and analysis. Descriptive statistics (frequencies and percentages) were done and the result was presented in the form of tables, figures, and text. Before any statistical analysis, the data were weighted to restore representativeness and to obtain a reliable estimate and standard error. A multilevel binary logistic regression analysis was applied because the DHS data has a hierarchical structure that violates the independent assumptions of the standard logistic regression model. To determine whether there was clustering or not, the Interclass Correlation Coefficient (ICC), Proportional Change in Variance (PCV), and Median Odds Ratio (MOR) were computed. In our study, four models were fitted; the null model (a model without explanatory factors), model I (a model with individual-level variables), model II (a model with community-level variables), and model III (a model with both individual and community-level variables). Model comparison was employed based on deviance (− 2 log-likelihood). Model III was chosen as the best-fit model since it had the lowest deviance among all models. Bivariable and multivariable multi-level logistic regression analyses were employed using the best-fitted model. In the bivariable analysis, variables having a *P*-value of less than 0.2 were selected for multivariable analysis. Lastly, variables with a *P*-value of < 0.05 in the multivariable analysis were declared as significant factors associated with HIV testing among men, and the adjusted odds ratio (AOR) with the 95% confidence interval (CI) was computed to determine the strength and direction of the associations.

## Results

### Socio-demographic characteristics of participants

A total weighted sample of 113, 270 men (15-64 years) were included in this study. About 22.4% of participants were in the age group of 15-19 years and nearly half (49.9%) of the study participants were married. Regarding residence, about 30.7% of the study participants were residing in urban settings. Concerning HIV knowledge status, nearly half (51.7%) of participants had comprehensive HIV/AIDS knowledge. Regarding stigmatized attitudes, most (95.6%) of participants had a negative attitude towards people with HIV/AIDS. About 64.8% of the study participants had no risky sexual behavior **(**Table 2**)**.

**Table 2:** Socio-demographic characteristics of participants in Eastern Africa (*N* = 113,270)

Variable	Frequency	Percent
Age in years		
15-19	25,355	22.4
20-29	34,786	30.7
30-39	26,633	23.5
40 and above	26,496	23.4
Marital status		
Never in union	44,992	39.7
Married	56,475	49.9
Living with partners	6858	6.0
Widowed, divorced, or separated	4845	4.4
Residence		
Urban	34,809	30.7
Rural	78,461	69.3
Age at first sex (years)		
Less than 20	81,474	71.9
20 and above	31,796	28.1
Community illiteracy level		
Low	56,394	49.8
High	56,876	50.2
Community poverty level		
Low	56,972	50.3
High	56,298	49.7
HIV knowledge		
Low	9183	8.1
High	45,481	40.1
Comprehensive	58,606	51.8
Stigma indicator		
No stigma	5019	4.4
Low	34,762	30.7
Moderate	58,005	51.2
High	15,484	13.7
Risky sexual behavior		
No risk	73,419	64.8
Some risk	31,737	28.0
High risk	8114	7.2
Covered by health insurance		
No	97,912	86.4
Yes	15,358	13.6
Sex of household head		
Male	95,428	84.3
Female	17,842	15.7

### Prevalence of HIV testing in Eastern Africa

**Fig. 1:**
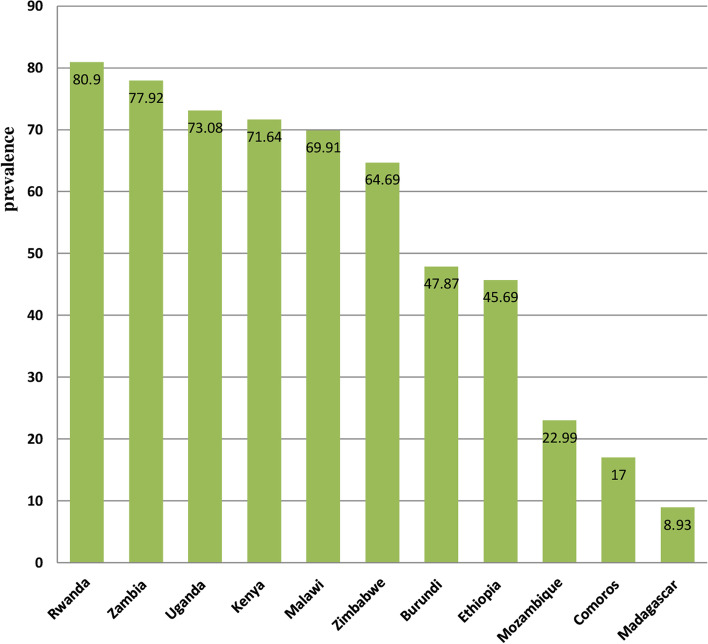
The prevalence of HIV testing among men in Eastern Africa

The overall prevalence of HIV testing among men in eastern Africa was 60.5% (95%CI: 60.2, 60.7%), ranging from 8.9% in Madagascar to 80.9% in Rwanda **(**Fig. 1**)**.

### Random Effect Model and Model Fitness

To determine the random-effect model, we used ICC, MOR, and PCV values. The ICC value of 0.059 in the null model indicates that 5.9% of the total variation in HIV testing coverage among men was due to cluster variability. Also, the MOR value of 1.24 in the null model indicates significant clustering of HIV testing among sexually active men happened. Moreover, the PCV (0.14) in the final model (model III) revealed that both the individual and community-level variables contributed approximately 14% of the variation in HIV testing. Since the final model which contains the individual level and community level factors had the lowest deviance, it was the best-fitted model **(**Table 3**).**

**Table 3:** Random effect model and model fitness for the assessment of HIV testing among men in Eastern Africa

Parameter	Null model	Model I	Model II	Model III
Interclass correlation coefficient (ICC)	0.0597225	0.0665384	0.052394	0.0644185
Percentage change in variation (PCV)	Ref	0.12	0.13	0.14
Median odds ratio (MOR)	1.24	1.31	1.15	1.29
Model comparison				
Log likelihood (LL)	−74,686.586	−63,379.705	−73,602.45	−62,825.153
Deviance	149,373.172	126,759.41	147,204.9	125,650.306

### Factors Associated with HIV Testing Among Men in Eastern Africa

To identify factors associated with HIV testing and counseling, we run the final model analysis (the best-fitted model) because it had the lowest deviance. In the multivariable multilevel analysis: the age of participants, marital status, residence, age at first sex, community poverty level, community illiteracy level, HIV knowledge, HIV stigma index, risky sexual behavior, and health insurance were factors significantly associated with HIV testing among men. Men in the age group between 20 and 29 (adjusted odds ratio (AOR) =2.51: 95% CI; 2.40, 2.62), 30–39 (AOR = 2.37:95% CI; 2.24, 2.51), and 40 and above years (AOR = 1.73:95% CI; 1.63, 1.83) had a higher chance of being tested for HIV than those with 15–19 years old. Regarding marital status, the odds of testing for HIV were 2.30 (AOR = 2.30: 95% CI; 2.19, 2.41), 1.60 (AOR = 1.60: 95% CI; 1.50, 1.72), and 1.62 (AOR = 1.62: 95% CI; 1.50, 1.75) times higher for men with married, living with partners, and widowed/divorced/separated, respectively, compared to men with unmarried. Concerning residence, men residing in rural settings had a 43% (AOR = 0.57: 95% CI; 0.55, 0.59) lower chance of being tested for HIV than their counterparts. Participants who had started sex after 20 years had a 15% (AOR = 0.85: 95% CI; 0.83, 0.86) lower chance of being tested for HIV compared with their counterparts. Participants from higher community illiteracy levels had 26% (AOR = 0.74: 95% CI; 0.69, 0.80) lower odds of being tested for HIV than their counterparts. Being from communities with higher community poverty levels had a 1.10 (AOR = 1.10: 95% CI; 1.03, 1.18) times higher odds of being tested for HIV than those with lower community poverty levels. Regarding HIV knowledge levels, participants who had high (AOR = 2.91: 95%CI: 2.75, 3.01) and comprehensive knowledge (AOR = 3.78: 95% CI; 3.57, 4.00) about HIV/AIDS were more likely to be tested for HIV than those with low HIV knowledge. Interestingly, men with low, moderate and high stigma scores had 8% (AOR = 0.92: 95% CI; 0.86, 0.99), 40% (AOR = 0.60: 95% CI; 0.56, 0.65), and 80% (AOR = 0.20: 95% CI; 0.18, 0.22) lower chance of being tested for HIV than those with no stigma. The odds of being tested for HIV were 1.71 (AOR = 1.71: 95% CI; 1.65, 1.76) and 2.41 (AOR = 2.41: 95% CI; 2.28, 2.56) times higher for men with some and high risky sexual behavior, respectively than those with no risky sexual behavior. Moreover, men who had health insurance coverage were 2.41 (AOR = 2.41: 95% CI; 2.30, 2.53) more likely to be tested for HIV than their counterparts **(**Table 4**).**

**Table 4:** The bivariable and multivariable multilevel binary logistic regression analysis of factors associated with HIV testing among men in Eastern Africa

Variable	Ever tested for HIV		COR (95% CI)	AOR (95% CI)
	Yes	No		
**Age in years**				
15-19	8830	16,525	1	1
20-29	23,324	11,463	3.98 (3.84, 4.12)*	2.51 (2.40, 2.62)*
30-39	19,048	7585	4.99 (4.81, 5.19)*	2.37 (2.24, 2.51)*
40 and above	17,282	9213	3.71 (3.58, 3.86)*	1.73 (1.63, 1.83)*
**Marital status**				
Never in union	21,438	23,554	1	
Married	39,261	17,214	2.65 (2.44, 2.57)*	2.30 (2.19, 2.41)*
Living with partners	4502	2356	2.10 (1.99, 2.22)*	1.60 (1.50, 1.72)*
Widowed/divorced/ separated	3282	1663	2.13 (2.01, 2.27)*	1.62 (1.50, 1.75)*
**Residence**				
Urban	24,841	9968	1	1
Rural	34,819	43,642	0.49 (0.47, 0.51)*	0.57 (0.55, 0.59)*
**Age at first sex (years)**				
Less than 20	46,068	35,406	1	1
20 and above	22,415	9381	0.82 (0.80, 0.84)*	0.85 (0.83, 0.86)*
**Community illiteracy level**				
Low	35,226	21,168	1	1
High	33,257	23,619	0.72 (0.68, 0.77)*	0.74 (0.69,0.80)*
**Community poverty level**				
Low	35,406	21,566	1	1
High	33,077	23,221	0.85 (0.79, 0.90)*	1.10 (1.03, 1.18)*
**HIV knowledge**				
Low	2365	6818	1	1
High	25,812	19,669	3.77 (3.58, 3.97)*	2.91 (2.75, 3.01)*
Comprehensive	40,306	18,300	6.41 (6.09, 6.75)*	3.78 (3.57, 4.00)*
**Stigma indicator**				
No stigma	3496	1523	1	1
Low	23,954	10,808	0.94 (0.88, 1.00)	0.92 (0.86, 0.99)*
Moderate	36,170	21,835	0.67 (0.63, 0.72)*	0.60 (0.56, 0.65)*
High	4863	10,621	0.18 (0.17, 0.20)*	0.20 (0.18, 0.22)*
**Risky sexual behavior**				
No risk	41,940	31,479	1	1
Some risk	20,667	11,070	1.39 (1.36, 1.43)*	1.71 (1.65, 1.76)*
High risk	5876	2238	2.05 (1.95, 2.16)*	2.41 (2.28, 2.56)*
**Covered by health insurance**				
No	56,573	41,339		1
Yes	11,910	3448	2.57(2.46, 2.68)*	2.41 (2.30, 2.53)*
**Sex of household head**				
Male	59,299	36,129	1	1
Female	9184	8658	0.63 (0.61, 0.65)*	0.96 (0.92, 1.01)

*P-value ≤0.05

## Discussion

Despite all efforts made, the prevalence of HIV testing in Eastern Africa remains low but indicates a tendency to improve [14]. The pooled prevalence of HIV testing among men in Eastern Africa was 60.5% (95% CI: 60.2, 60.7%). The present finding was higher than the study conducted in different countries [1, 4, 7, 12, 21, 22]. However, the estimated prevalence of HIV testing in this study was lower than the findings from the survey done in Cambodia [17]. Similarly, it is also lower than the study conducted in Uganda [3]. The possible explanation for this difference could be the variation in lifestyle and cultural beliefs among different countries. Besides, the differences in quality and availability of HIV testing services, and knowledge about HIV/AIDS could be the possible reason for the variation in the prevalence of HIV testing across different countries [7].

Our findings indicated that age, marital status, residence, age at first sex, community poverty level, community illiteracy level, HIV knowledge, HIV stigma index, risky sexual behavior, and health insurance were significantly associated with the uptake of HIV testing services among men in Eastern Africa. Respondents’ age was an important factor significantly associated with HIV testing among men. Older men aged 20 years and above had higher odds of being tested for HIV than adolescent men aged 15–19 years. This finding is supported by previous studies done in Haiti [22], South Africa [23], Malawi [7], and Ethiopia [12]. The low levels of HIV testing among adolescent men might be due to several factors. Firstly, adolescent men might have low knowledge about HIV/AIDS and are inaccessible to health care services. For example, in a previous report, less than 20% of adolescent men aged between 15 and 19 years in Africa know their HIV status and 90% of HIV adolescents in the world live in sub-Saharan Africa [24]. Secondly, adolescent men aged 15-19 years may have limited sexual experiences and are not informed about sexual issues; thus perceive themselves as having a lower risk of HIV, this in turn contributes to low uptake of HIV testing in this age group. Thirdly, fear and stigmatized attitude towards HIV/AIDS can also reduce the uptake of HIV testing among adolescent men [7].

The findings further reveal that HIV testing among men was significantly associated with marital status. The odds of HIV testing were higher among married men compared to those with never been in a union. The result was in line with the studies conducted elsewhere [7, 12, 25, 26]. This could be due to married men may have a great sense of family responsibility than single men. Another reason for this might be couple’s discussion about HIV to protect their marriage and future children’s health.

In this study, men from rural areas had a lower chance of being tested for HIV, which is consistent with the studies conducted in Malawi [7] and Ethiopia [27, 28]. This could be because HIV testing services are more readily available and accessible in urban areas than in rural areas [29, 30]. Men who had started sex after 20 years of age had a lower chance of being tested for HIV compared with their counterparts, which is supported by the study carried out in Malawi [7] and Ethiopia [31]. This is justified by being young age at first sexual intercourse is correlated with a higher risk of getting various sexually transmitted diseases and engaging in risky sexual practices that could lead to HIV infection, forcing them to know their HIV status [32].

In the present study, community illiteracy level was significantly associated with ever being tested for HIV. Men from higher community illiteracy levels had lower chances of being tested for HIV than their counterparts, and this is in agreement with the reports conducted elsewhere [33, 34]. This might be justified by educated men may be more exposed to HIV/AIDS-related knowledge, have a better understanding about the importance of HIV testing, and ability to make better decisions about HIV testing. Besides, educational attainment may contribute to greater awareness about the importance of knowing an individual’s HIV status, which could lead to increased uptake of HIV testing [35, 36]. Being from higher community poverty levels had a higher likelihood of being tested for HIV than their counterparts and this finding is contrary to a study conducted in Ethiopia [1]. The possible explanation for this may be due to having higher socioeconomic status may be associated with a greater awareness of risks and with decreased financial barriers to HIV testing, which in turn reduced the uptake of HIV testing [34]. The study revealed that men with higher and comprehensive knowledge about HIV had a greater likelihood of being tested for HIV compared with low knowledge. This finding is consistent with the result of previous studies [12, 28, 31, 36]. This is justified by men having comprehensive HIV knowledge that may be associated with a good understanding of the potential risks of HIV, and the need to be tested to know their status to prevent the disease and its complications. Interestingly, men with higher stigma scores had a lower chance of being tested for HIV than those with no stigma. Previous studies had also indicated participants with higher HIV stigma scores had lower odds of being tested for HIV [1, 37]. The possible explanation for this could be participants may be hesitant to test since the disclosure of a positive HIV test result can result in loss of friendship, family relationships, jobs, and housing and health care as a result of discrimination [38, 39].

In this study, men who had risky sexual behavior had higher odds of being tested for HIV compared with their counterparts. This is in agreement with another study [20]. This might be due to participants with risky sexual behavior may have frequent fear and uncertainty about their HIV serostatus and are usually suspicious and worried that they have been infected with HIV. This motivates individuals to seek voluntary counseling and testing services regularly [40]. Moreover, being covered by health insurance was more likely to be tested for HIV compared with their counterparts. This finding is supported by the results of other studies [7, 12, 22]. This is explained as men with health insurance are more likely to visit health care facilities to seek health services including HIV testing since the services are free.

### Strength and limitations of the study

As strength, this study was based on the weighted large sample size of nationally representative data drawn in eleven East African countries. Besides, we used the multilevel analysis to adjust the hierarchical nature of the DHS data. Furthermore, because the findings were based on the national survey data, they can provide important information for program managers and policymakers to develop good interventions at the regional and national levels. This study had some limitations: Firstly, since the DHS survey was dependent on participants’ self-reports, it could lead to recall bias. Secondly, since this analysis was based on the cross-sectional nature of DHS collected data, it is impossible to indicate the temporal relationship between outcome and explanatory variables. Lastly, the use of old data sets from Madagascar may scale up of access to HIV testing service.

## Conclusion

The pooled prevalence of HIV testing among men in Eastern Africa was relatively higher than the findings from the previous studies. In the multivariable multilevel analysis: Age of participant, marital status, residence, age at first sex, community poverty level, community illiteracy level, HIV knowledge, HIV stigma indicator, risky sexual behavior, and health insurance were factors significantly associated with HIV testing among men. Therefore, all the concerned bodies need to develop an integrated strategic plan through providing special attention to the factors that affect the uptake of HIV testing to raise awareness about the importance of HIV testing and to prevent HIV/AIDS transmission.

## Data Availability

Data is available online and you can access it from www.measuredhs.com.

## Abbreviations

AIDS: Acquired Immune-Deficiency Syndrome
CI: Confidence Interval
DHS: Demographic Health Survey
ICC: Intraclass Correlation Coefficient
HIV: Human-Immunodeficiency Virus
LL: Likelihood
MOR: Median Odds Ratio
PCV: Proportional Change in Variance
WHO: World Health Organization
